# Dr. Jeremiah Hubert Kelly

**Published:** 1910-04-16

**Authors:** 


### Obituary.

The death is announced (at Blackrock, Co. Dublin) of Dr. Jeremiah Hubert Kelly, M.D., in his ninety-third year. He became an L.R.C.S. of Ireland in 1838, taking the L.M. after study at the Lying-in Hospital in the same year. In 1839 he graduated M.D. at Glasgow.

